# Attractive Sugar Bait Formulation for Development of Attractive Toxic Sugar Bait for Control of *Aedes aegypti* (Linnaeus)

**DOI:** 10.1155/2022/2977454

**Published:** 2022-06-18

**Authors:** Sarita Kumar, Aarti Sharma, Roopa Rani Samal, Manoj Kumar, Vaishali Verma, Ravinder Kumar Sagar, ShriPati Singh, Kamaraju Raghavendra

**Affiliations:** ^1^Department of Zoology, Acharya Narendra Dev College, University of Delhi, Kalkaji, New Delhi 110 019, India; ^2^National Institute of Malaria Research (ICMR), Sector 8, Dwarka, New Delhi 110 077, India; ^3^Present Address - H.No. 28B, Block ED, Pitampura, Delhi 110 088, India

## Abstract

**Background:**

Attractive toxic sugar bait (ATSB), based on “attract and kill” approach, is a novel and promising strategy for mosquito control. Formulation of an attractive sugar bait (ASB) solution by selecting an efficient olfaction stimulant and preparation of an optimized sugar-attractant dosage is a significant component for the success of the approach.

**Methods:**

Current study evaluated relative potential of nine ASBs, formulated by combination of sugar and fresh fruit juices (guava, mango, muskmelon, orange, papaya, pineapple, plum, sweet lemon, and watermelon) in attracting *Aedes aegypti* adults. Freshly extracted and 48-hour-fermented juices were combined with 10% sucrose solution (w/v) in 1 : 1 ratio. Cage bioassays were conducted against two laboratory strains (susceptible: AND-*Aedes aegypti*; deltamethrin-selected: AND-*Aedes aegypti*-DL10) and two field-collected strains (Shahdara strain of *Aedes aegypti*: SHD-Delhi; Govindpuri strain of *Aedes aegypti*: GVD-Delhi). Each of the nine ASBs was assayed, individually or in groups of three, for its attraction potential based on the relative number of mosquito landings. The data were analysed for statistical significance using PASW (SPSS) software 19.0 program.

**Results:**

The prescreening bioassay with individual ASB revealed significantly higher efficacy of ASB containing guava/plum/mango juice than that containing six other juices (*p* < 0.05) against both the laboratory and field strains. The bioassay with three ASBs kept in one cage, one of the effective ASBs and two others randomly selected ASBs, also showed good attractancy of the guava/plum/mango juice-ASB (*p* < 0.05). The postscreening assays with these three ASBs revealed maximum attractant potential of guava juice-sucrose combination for all the four strains of *Ae. aegypti. Conclusion*. Guava juice-ASB showed the highest attractancy against both laboratory and field-collected strains *of Ae. aegypti* and can be used to formulate ATSB by combining with a toxicant. The field studies with these formulations will ascertain their efficacy and possible use in mosquito management programs.

## 1. Introduction

*Aedes aegypti* (Linnaeus) is the major insect disease vector for transmitting viral diseases, dengue, yellow fever, Chikungunya, and Zika [1]. In 2019, the largest number of dengue cases (5.2 million) were ever reported worldwide affecting all WHO regions [2]. Since the use of chemical-based control interventions is associated with environmental concerns, there is a need for natural and environmentally safe interventions [3].

Insecticide-laced attractive toxic sugar bait (ATSB) is considered a new mosquito control method based on the feeding behaviour of mosquitoes [4]. The mosquitoes feed on plant sugars (sucrose and fructose), nectar, honeydew, etc., as a source of energy for their nutrition and survival [5] and use visual and olfaction cues to locate flowers and fruits [6]. Sugar-seeking mosquitoes are attracted by the volatile components of fruits and flowers [7], damaged and rotted fruits [8], and various fruits, edible seeds, flowers, and insect honeydew [9]. Thus, a combination of sugar, an attractant derived from these natural plant sources, and an insecticide can be an efficient control method to attract and kill the mosquitoes. The sugar-attractant-insecticide mixture (known as ATSB) has been used successfully as a bait for attraction of mosquitoes in the laboratory experiments [10, 11] and also in field conditions [12–14] and showed effective toxic attractant potential resulting in high rates of mosquito mortality. It has been demonstrated that ATSB can be utilized as bait stations indoors and as spray outdoors on vegetation [15] and was found to be an effective alternative tool for the present synthetic chemical interventions [16].

The foremost requirement to formulate an effective ATSB is to select an effective olfaction stimulant-sugar mixture, which can attract mosquitoes, followed by identification of optimum dosage of a toxicant to kill the attracted mosquitoes. The present study is an attempt to formulate an attractive sugar bait (ASB) comprising a mixture of fruit juice as an attractant and 10% sucrose solution as a sugar food source. Nine fruit juices were used to prepare nine different ASBs on the basis of their local availability and reports in the literature. Individual juice-sugar mixture was screened against two laboratory strains (one susceptible and one deltamethrin-selected) and two field-collected strains of *Ae. aegypti*. Based on the results, extensive work is being carried out in the laboratory to formulate an effective ATSB using deltamethrin and dinotefuran as toxicants. The screening assays have been conducted with different ratios and dosages of the components used in ATSB to identify an effective formulation. The results are encouraging, and the field assays with the formulation will ascertain its efficacy against mosquitoes. This will facilitate the application of a suitable ATSB outdoors as a probable mosquito management strategy in conjunction with existing vector control interventions.

## 2. Materials and Methods

### 2.1. Rearing of *Aedes aegypti* Mosquitoes

A total of four strains of *Ae. aegypti*, two laboratory strains and two field strains, were colonized in the Insect Pest and Vector Control Laboratory, Acharya Narendra Dev College, University of Delhi, India.

### 2.2. Laboratory Strains

1. Insecticide susceptible strain of *Ae. aegypti* (AND- *Aedes aegypti* ) established in the year 2009 without any selection pressure of any insecticide (susceptibility to deltamethrin: LC_50_ = 0.00082 mg/L).
2. Deltamethrin-selected strain of *Ae. aegypti* (AND-*Aedes aegypti*-DL10) subjected to deltamethrin selection pressure at larval stage at LC_90_ level for 10 successive generations and kept under constant selection pressure (susceptibility to deltamethrin: LC_50_ = 0.00813 mg/L, resistance ratio (RR) = 9.91).

### 2.3. Field Strains

1. Shahdara strain of *Ae. aegypti* (SHD-Delhi) was collected from the Shahdara locality of the East Delhi (28.689°N, 77.290°E) in May 2021 (susceptibility to deltamethrin: LC_50_ = 0.01705 mg/L, resistance ratio (RR) = 20.79).
2. Govindpuri strain of *Ae. aegypti* (GVD-Delhi) was collected from the Govindpuri locality of the South East Delhi (28.534°N, 77.265°E) in the month of June 2021 (susceptibility to deltamethrin: LC_50_ = 0.00680 mg/L, resistance ratio (RR) = 8.29).

The mosquito culture was maintained under controlled conditions of temperature (27 ± 2°C), relative humidity (80 ± 10%), and light-dark photoperiod (14 : 10). Adult mosquitoes were kept in screened cloth cages (45 × 40 × 40 cm) and provided with 10% sucrose solution soaked in cotton as a source of food. The blood meal from albino mice was given to female mosquitoes on alternate days. A bowl filled with dechlorinated water lined with filter paper strips was kept inside the cage for the collection of eggs. The egg-laden strips were transferred in an enamel/plastic tray (25 × 30 × 5 cm) filled with 1.5–2.0 L of dechlorinated water for hatching. The larvae were fed on the mixture of finely ground dog biscuits and yeast (3 : 2 w/w). The water was changed each day to avoid the formation of any scum/froth on the water surface. The pupae were separated regularly and kept in the cloth cages for adult emergence [17, 18]. The two- to three-day-old, 24 h starved, nonblood fed emerged adults were used for the studies to assess their attractant potential towards different ASBs.

### 2.4. Preparation of Attractive Sugar Bait (ASB)

Attractive sugar bait (ASB) was prepared by mixing a fruit juice as an attractant and 10% sucrose solution as a sugar food source. Nine fruits—guava (*Psidium guajava*), mango (*Mangifera indica*), muskmelon (*Cucumis melo*), orange (*Citrus sinensis*), papaya (*Carica papaya*), pineapple (*Ananas comosus*), plum (*Prunus domestica*), sweet lemon (*Citrus limetta*), and watermelon (*Citrullus lanatus*)—were selected on the basis of their local availability and reports in similar trials elsewhere with a potential to attract mosquitoes [9, 19]. The fruits were peeled, and the fruit pulps were homogenized separately using a mixer grinder (Sujata Mixer, Powermatic Plus, India). The extracted juices kept in a closed container were left in the laboratory at ambient temperature for 48 h fermentation to enhance their olfaction potential by increasing volatile components. Nine different ASBs were formulated by combining each fermented juice and 10% sugar solution (w/v) in 1 : 1 proportion. The sucrose solution (10% w/v) in water was used as control in the assays.

### 2.5. Attractive Sugar Bait (ASB) Cage Bioassay

#### 2.5.1. Prescreening Bioassay

**Figure 1:**
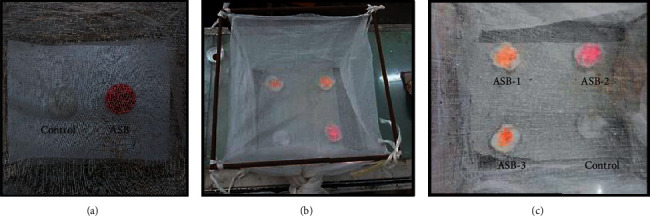
Cage bioassay with *Aedes aegypti* adults (*n* = 50, 25 males and 25 females). (a) Prescreening cage with a control (10% sucrose solution) and one ASB placed at two sides. (b) Screening cage with three ASBs (e.g., ASBs 1, 2, and 3) and a control (10% sucrose solution) bait placed in four corners. (c) Inside view of screening cage with three ASBs and a control.

Prescreening cage bioassays were conducted with each of the nine ASBs. Eighteen cotton discs of same weight (0.5 g) and size were taken. Nine discs (experimental) were soaked separately with 5 mL of nine ASBs, and nine cotton discs (control) were soaked in 5 mL of sucrose solution taking care to avoid excess dripping. One of the experimental discs and one control disc were placed at the two sides of a screened cloth cage (45 × 40 × 40 cm) (Figure 1(a)).

Twenty-five males and 25 females of *Ae. aegypti* were introduced into each cage. The landing counts on the cotton discs were recorded at an interval of every ten minutes for one hour, or until the landing of mosquitoes ceased. The positions of the cotton discs containing ASBs and controls were changed every 10 minutes of intervals to negate the position effect. Four replicates were conducted to identify the attractancy of each ASB, making a total of 200 adults for each assay. The assays were carried out on all the four strains: two laboratory strains (AND*-Aedes aegypti* and AND*-Aedes aegypti*-DL10) and two field strains (*Aedes aegypti* (SHD-Delhi) and *Aedesaegypti* (GVD-Delhi)).

#### 2.5.2. Screening Bioassay

The screening bioassay was conducted in three separate cages (A, B, and C) by placing three individual ASBs cotton discs and one control cotton disc at the four corners of each cloth cage (Figures 1(b) and 1(c)). The three ASB discs comprised one of the effective ASBs obtained in the prescreening assay and two other randomly selected ASBs. The ASBs used in respective cages were as follows:

  Cage A: watermelon juice-ASB, muskmelon juice-ASB, and mango juice-ASB
  Cage B: papaya juice-ASB, orange juice-ASB, and plum juice-ASB
  Cage C: pineapple juice-ASB, sweet lemon juice-ASB, and guava juice-ASB

The assay was conducted as in the prescreening, and average landing counts on each ASB were analysed statistically. The percentage of mosquitoes landed on each ASB was calculated by dividing the number of mosquitoes landed by total number of mosquitoes released in the cage. The significant differences among the means of control and different ASBs were calculated with *p* < 0.05 considered as significant value.

#### 2.5.3. Postscreening Bioassay

Three ASBs which showed significantly higher attractancy for mosquitoes in the prescreening and screening bioassays than other ASBs (*p* < 0.05) were selected for the postscreening assay to estimate the relative mosquito attractancy. The postscreening cage bioassay was conducted by placing three cotton discs containing these three ASBs at three corners and the control cotton disc at the fourth corner of a cage.

The average landing counts on each of the three ASBs were scored as described earlier, and the data were analysed statistically. An attraction index (mean number of mosquitoes attracted to the baits/mean number of mosquitoes attracted to the control) was calculated to compare the relative attraction potential of the baits for the mosquitoes.

### 2.6. Statistical Analysis

The average landing counts on each ASB were analysed statistically using PASW (SPSS) software 19.0 program. The standard error of mean (SEM) was calculated, and the data were subjected to one-way ANOVA followed by Tukey's all pairwise multiple comparison test in order to determine the significant differences among the means of control and different ASBs. The *p* value less than 0.05 was considered as significant.

## 3. Results

### 3.1. Prescreening Bioassay

The number of mosquitoes of the laboratory and field strains of *Ae. aegypti* attracted towards individual juice-sugar mixtures (ASBs) in relation to the respective control is presented in Table 1. The plum juice-ASB showed highest attracting potential towards AND-*Aedes aegypti* strain and both the field-collected *Aedes aegypti* (SHD-Delhi) and *Aedes aegypti* (GVD-Delhi) strains, followed by guava juice-ASB and mango juice-ASB. However, for AND*-Aedes aegypti-*DL10, guava juice-ASB showed maximal attraction followed by plum juice-ASB and mango juice-ASB.

### 3.2. Screening Bioassay

**Figure 2:**
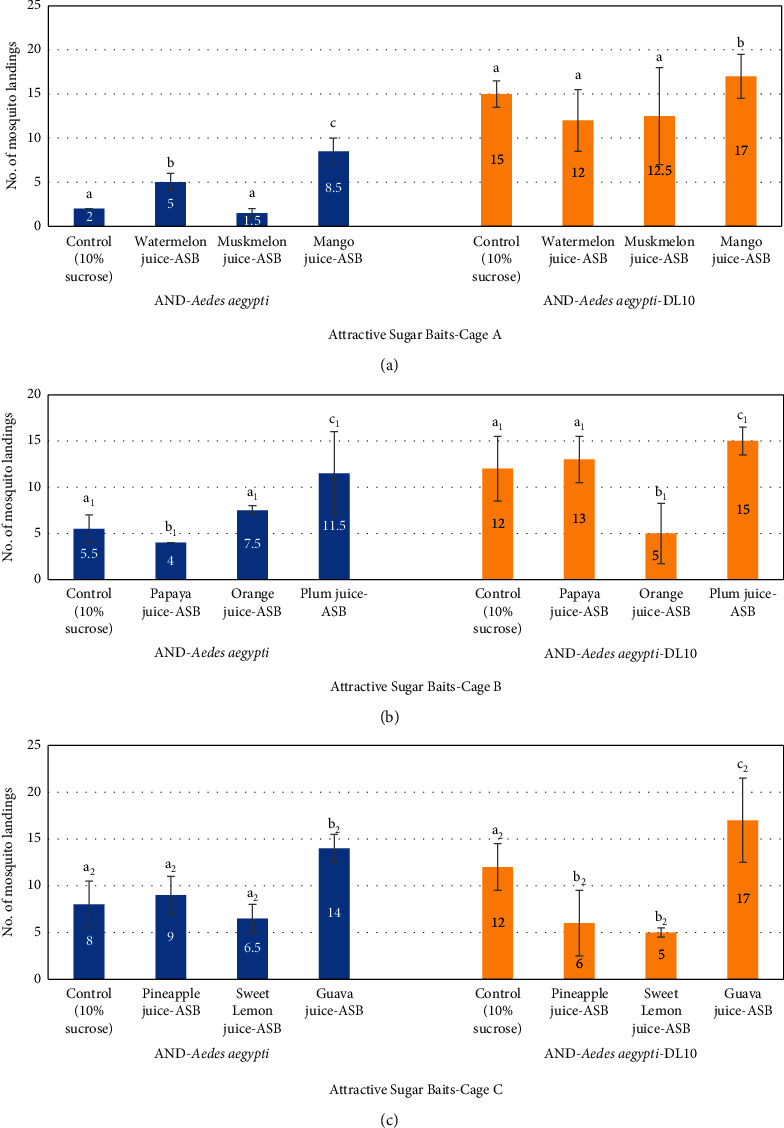
Screening assay showing number of landings in the laboratory population of AND-*Aedes aegypti* and AND-*Aedes aegypti*-DL10 on three ASBs placed along with a control in one of the cage.

The screening assays against laboratory strains of *Aedes aegypti* (AND-*Aedes aegypti* strain and AND-*Aedes aegypti*-DL10) and statistical results with three efficient ASBs, mango juice-ASB, plum juice-ASB and guava juice-ASB, kept, respectively, in cages A, B, and C, each with randomly selected two other ASBs and a control bait are presented in Figure 2. The percentage of landings of AND-*Aedes aegypti* adult mosquitoes on nine different ASBs was calculated and was found in the range of 3–28%, while landings on the control bait was recorded 4–16%. On the other hand, the percentage of landings observed in AND-*Aedes aegypti*-DL10 was 10–34% in comparison to 24–30% on control bait (Table 2).

Among all the nine ASBs tested in three cages, guava juice-ASB showed the highest attractancy (14 landings), followed by plum (11.5) and pineapple juice (9) against AND-*Aedes aegypti*. However, less mosquitoes landed on mango, orange, sweet lemon, watermelon, papaya, and muskmelon-ASBs, and the landings were in the range of 8.5–1.5 (*p* > 0.05). The landings on sugar control discs were, nevertheless, in the range of 2–8 in all the three cages. When all the nine juices served as baits to check their attractancy against AND-*Aedes aegypti*-DL10, maximum landing of mosquitoes was observed on guava and mango juice-ASB's (17 each), followed by plum juice-ASB (15). The mosquito landings were recorded in the range of 13 to 5 for other 6 juices, while for control, it was 12–15.

**Figure 3:**
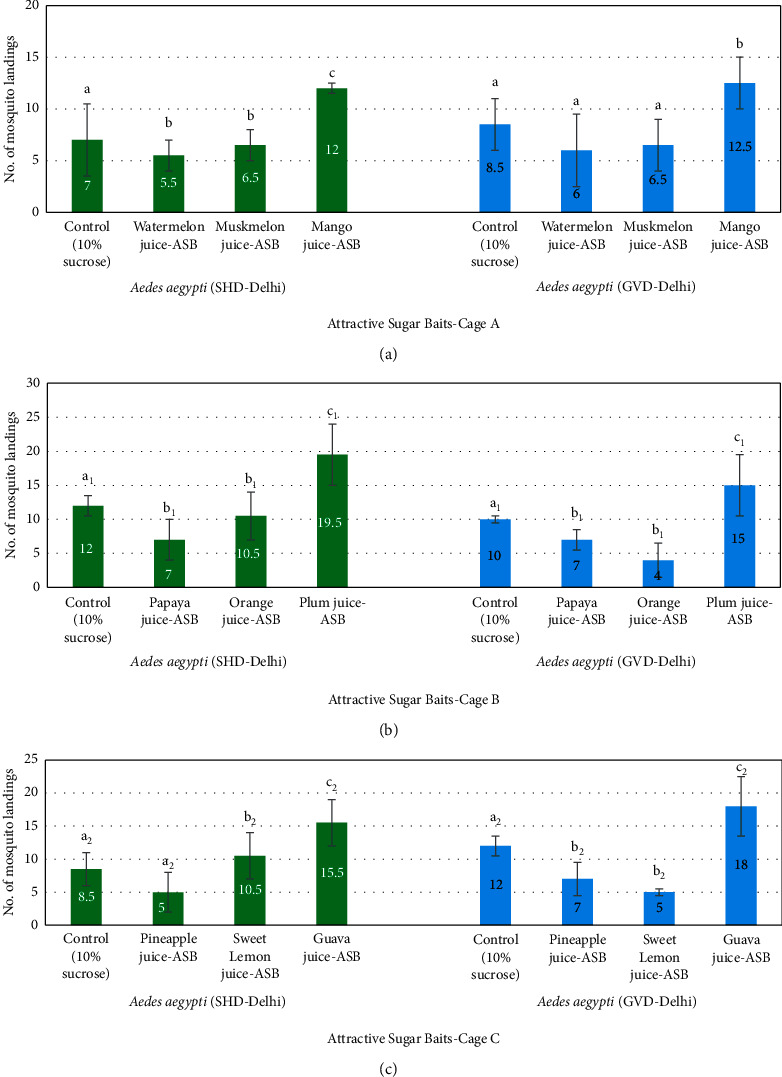
Screening assay showing number of landings by the field population of *Aedes aegypti* (SHD-Delhi) and *Aedes aegypti* (GVD-Delhi) on different ASBs placed along with a control in one cage.

The screening assays conducted against *Aedes aegypti* (SHD-Delhi) strain and *Aedes aegypti* (GVD-Delhi) strain with mango juice-ASB, plum juice-ASB, and guava juice-ASB kept in respective cages A, B, and C, each along with randomly selected two ASBs and a control bait, showed results similar to the laboratory strains (Table 2 and Figure 3). The tests showed 10–39% landings of *Aedes aegypti* (SHD-Delhi) strain mosquitoes on the 9 juice-ASBs, while control bait attracted 14–24% mosquitoes. The average mean value of number of mosquitoes that landed on different ASBs was recorded the highest for plum juice-ASB (19.5), followed by guava (15.5) and mango juice-ASBs (12), while it was the lowest for pineapple juice-ASB (5). The values of the 3 juice-ASBs with maximum attraction potential showed significant differences with other juices (*p* < 0.05).

When these 9 juices were used as ASBs against mosquitoes of *Ae. aegypti* (GVD-Delhi) strain, the percentage of landings recorded was in the range of 8–36% in the screening test while on controls they were in the range of 17–24%. Almost similar observations were noted with the SHD-Delhi strain. The average landings of mosquitoes on guava juice-ASB were 18 and highest among all the 9 ASBs, followed by plum and mango juices-ASBs that recorded 15 and 12.5 landings, respectively, while orange juice recorded the least attractant potential (4).

### 3.3. Postscreening Bioassay

**Figure 4:**
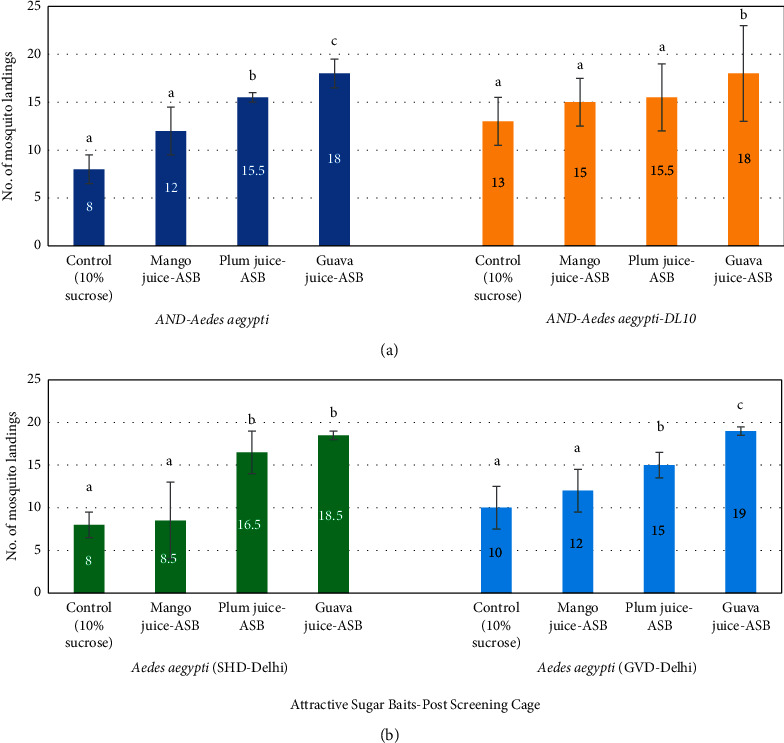
Postscreening assay showing number of landings by laboratory (*AND-Aedes aegypti* and *AND-Aedes aegypti-DL10*) and field population *Aedes aegypti* (SHD-Delhi) and *Aedes aegypti* (GVD-Delhi) of the three most efficient ASBs placed along with a control in one cage.

Based on the results of screening tests, 3 ASBs containing mango, plum, and guava juices were selected and used in postscreening assays against both the laboratory and field-collected strains. Among the three juices, guava juice-ASB showed the maximum attraction potential, followed by plum juice-ASB and mango juice-ASB. The mean landings of the laboratory strains of *Ae. aegypti* was 18 on guava juice-ASB, 15.5 on plum juice-ASB, and in the range of 12–15 on mango juice-ASB (Figure 4).

In case of field-collected strains, the mean landings on guava juice-ASB were 19 for *Aedes aegypti* (GVD-Delhi) strain and 18.5 for *Aedes aegypti* (SHD-Delhi) strain. These values for plum juice-ASB were 16.5 for *Aedes aegypti* (SHD-Delhi) strain and 15 for *Aedes aegypti* (GVD-Delhi), while mango juice-ASB could attract only 8.5 and 12 mosquitoes, respectively (Figure 4).

When the data of these three ASBs were compared statistically, the guava juice-ASB showed significantly higher olfaction attraction stimulant than the rest two ASBs (*p* < 0.05).

### 3.4. Relative Attractant Potential of Selected ASBs against Laboratory and Field Strains

The relative attractant potential of the three ASBs assayed in postscreening tests, guava juice-ASB, plum juice-ASB, and mango juice-ASB, against both the laboratory strains and field strains of *Ae. aegypti* with respect to controls is presented in Table 3. Guava juice-ASB exhibited the highest relative attractancy towards three strains of *Ae. aegypti*: AND-*Aedes aegypti* (2.25), *Aedes aegypti* (SHD-Delhi) (2.31), and *Aedes aegypti* (GVD-Delhi) (1.90), followed by plum juice-ASB (1.94, 2.06, and 1.50) and mango juice-ASB (1.50, 1.06, and 1.20) against these three strains, respectively. However, AND-*Aedes aegypti-*DL10 strain showed maximum attraction for mango juice-ASB (1.54), followed by guava juice-ASB (1.38) and plum juice-ASB (1.19).

## 4. Discussion

*Aedes aegypti* (Linnaeus) is the major insect disease vector for transmitting viral diseases, dengue, yellow fever, Chikungunya, and Zika [1]. Application of chemical-based interventions has raised health and environmental concerns leading to the need of an eco-friendly control measure. ATSB is a new vector control paradigm based on the use of a combination of sugar as a source of energy, a fruit juice as an olfaction stimulant to attract the adults, and a toxicant to kill the attracted adults. The efficacy of ATSB, however, depends on the efficacy of the olfaction component to lure the adult mosquitoes in large numbers on the bait.

The present study was conducted to assess the relative attractant potential of nine ASBs prepared using nine fermented fruit juices: guava, mango, muskmelon, orange, papaya, pineapple, plum, sweet lemon, and watermelon, mixed individually with 10% sucrose (1 : 1). The ASB formulations were assayed against two laboratory strains (laboratory susceptible: AND-*Aedes aegypti* and deltamethrin-selected: AND-*Aedes aegypti*-DL10) and two field-collected strains (*Aedes aegypti* (SHD-Delhi) and *Aedes aegypti* (GVD-Delhi)). Prescreening of the individual nine ASBs against all the four strains showed that the ASBs containing guava juice, plum juice, and mango juice possessed significantly higher attractant potential against all the four strains with respect to the control (*p* < 0.05). In the screening assays, conducted with three ASBs kept in three corners of a single cage along with a control in the fourth corner, the maximum attractancy was of the guava juice-ASB than the other ASBs (*p* < 0.05) for the four laboratory and field strains of *Ae. aegypti*. The muskmelon and papaya juice-ASBs were found less efficient against AND-*Aedes aegypti* strain, orange and sweet lemon juice-ASBs against AND-*Aedes aegypti*-DL10, papaya and pineapple juice-ASB's against *Aedes aegypti* (SHD-Delhi), and orange and sweet lemon juice-ASBs against *Aedes aegypti* (GVD-Delhi). None of the juices used as baits was observed as repellent.

The guava juice has been reported to possess high attraction potential against different mosquito species. The field studies in Bandiagara district, Mali, investigated attractancy of 26 different local fruits and seedpods to pyrethroid-resistant *Anopheles gambiae* and demonstrated highest landings on guava fruit with mean landings of 14 by females (5.83 attraction index) and 9 by males (5.63 attraction index) [9]. The use of guava juice in the form of ASB in these fields could attract 101.6 ± 9.3 females and 55.9 ± 4.66 males of *An. gambiae* per light trap used in the fields [20]. The effective use of 48 h ripened guava juice in the ASB to attract *An. gambiae, An. arabiensis*, and *Culex quinquefasciatus* has been demonstrated in laboratory and field trials [19]. Hut trials with ATSB formulated with ASB-containing guava juice have been conducted in Côte d'Ivoire against *An. gambiae* based on the earlier results demonstrating its efficacy to attract mosquitoes [21].

In Bagamoyo, Tanzania, studies were conducted to attract *An. arabiensis* in semifield conditions with six ASBs formed with juices of guava (*Psidium guajava*), banana (*Musa*), mango (*Mangifera indica*), orange (*Citrus sinensis)*, tomato (*Solanum lycopersicum)*, watermelon (*Citrullus lanatus*), and papaya (*Carica papaya*), and showed maximum attractancy of orange juice-ASB (*n* = 337), followed by tomato (*n* = 318) and guava juice-ASBs (*n* = 315) [22]. The formulation of ASBs with a mixture of mango and guava juice (1 : 1) has been assessed for its possible use in ATSB against *Ae. albopictus* [23]. Similarly, the use of ASB containing the mixture of guava and honey melon juice could attract 28.3–53.1% females and 36.9–78.3% males of *An. gambiae* s.l. population [24].

The ASB formulated with a mixture of commercially available guava and mango juice (1 : 1) combined with brown sugar was found to be a promising lure for *Ae. albopictus* and *Ae. aegypti* to be used in ATSB in fields of Florida [23, 25]. In Brazil, the guava juice-ASB, mango juice-ASB, and cupuacu juice-ASB have been used to investigate their attractant potential against *Ae. aegypti* [26]. They reported maximum attractancy of male adults towards mango juice-ASB, followed by guava and cupuacu juice-ASBs while females were attracted more to guava juice-ASB, followed by cupuacu and mango juice-ASBs.

Based on the results obtained in the current study, an extensive work has been carried out in the laboratory to formulate an efficient ATSB to kill the attracted mosquitoes. The ASBs containing guava/plum juice are combined with deltamethrin, a pyrethroid insecticide or dinotefuran, a neonicotinoid insecticide. The adult susceptibility assays with the formulations prepared have been carried out, which have caused 85–95% mortality in an hour, indicating good efficacy of the formulated ATSBs against both the susceptible and resistant strains of *Ae. aegypti*. More ATSB formulations with varied dosages and proportion of deltamethrin/dinotefuran and other insecticides are tested against *Ae. aegypti* to determine their efficacy and obtain 100% adult mortality at the possible lowest dosage. The field studies with the optimized effective formulations will ascertain their efficacy and the possible use in mosquito management programs.

## 5. Conclusions

The results of the current study indicate the maximum attractant potential of guava juice-ASBs against laboratory susceptible strain and both the field strains of *Ae. aegypti* followed by plum juice-ASB and mango juice-ASB, while against deltamethrin-selected laboratory AND-*Aedes aegypti*-DL10 strain, mango juice-ASB showed the maximum attractancy. In comparison, rest of the six ASBs did not possess significant attractancy scoring low landings. Based on these results, the formulation of an efficient ATSB with guava/plum juice-ASB and deltamethrin/dinotefuran as the toxic component is under progress and we have obtained encouraging results. We propose that the application of ATSB outdoors could be employed as a plausible tool in mosquito management program in conjunction with existing vector control interventions indoors and outdoors as sugar feeding is an essential component of the adult mosquito's life. The approach may also have relevance for surveillance and control of other coinhabiting mosquitoes of *Ae. aegypti*.

## Figures and Tables

**Table 1:** Number of mosquitoes (laboratory strains and field strains) attracted towards different juice-sugar mixtures (ASBs) in prescreening cage bioassays.

S. No.	ASB	Laboratory strains				Field strains			
		AND*-Aedes aegypti*		AND*-Aedes aegypti*-DL10		*Aedes aegypti* (SHD-Delhi)		*Aedes aegypti* (GVD-Delhi)	
		No. of mosquitoes landed on the bait ± SEM	Control	No. of mosquitoes landed on the bait ± SEM	Control	No. of mosquitoes landed on the bait ± SEM	Control	No. of mosquitoes landed on the bait ± SEM	Control
1.	Water melon	5 ± 0.408 a^*∗*^	7.75 ± 0.629^#^	11.5 ± 1.190 a^*∗*^	12.5 ± 0.5^*∗*^	5.25 ± 0.629 a^*∗*^	8 ± 0.816^#^	5.5 ± 0.288 a^*∗*^	8 ± 0.707^#^

2.	Musk melon	5.25 ± 0.250 a^*∗*^	9.75 ± 0.853^#^	13 ± 0.816 a^*∗*^	14.25 ± 0.478^#^	6.25 ± 0.478 ab^*∗*^	10 ± 0.816^#^	6.25 ± 0.478 a^*∗*^	9.5 ± 0.288^#^

3.	Orange	5.75 ± 0.629 a^*∗*^	9.75 ± 0.478^#^	5.25 ± 0.25 b^*∗*^	10.25 ± 0.629^#^	11.25 ± 0.478 c^*∗*^	12 ± 0.408^*∗*^	4.75 ± 0.478 a^*∗*^	9.75 ± 0.478^#^

4.	Sweet lemon	7.25 ± 0.478 a^*∗*^	9.25 ± 0.478^#^	7.25 ± 0.478 c^*∗*^	10 ± 0.707^#^	11.25 ± 0.629 c^*∗*^	8.5 ± 0.645^#^	5.75 ± 0.478 a^*∗*^	10 ± 0.408^#^

5.	Papaya	4.5 ± 0.500 a^*∗*^	11.75 ± 0.629^*∗*^	11 ± 0.408 a^*∗*^	11.5 ± 0.500^*∗*^	8.25 ± 0.478 b^*∗*^	13.25 ± 0.750^#^	5.75 ± 0.750 a^*∗*^	11.5 ± 0.288^#^

6.	Mango	10 ± 0.577 b^*∗*^	8.5 ± 0.500^#^	17.25 ± 0.853 d^*∗*^	9.75 ± 0.629^#^	12 ± 0.577 c^*∗*^	8.5 ± 0.645^#^	11.5 ± 0.645 b^*∗*^	8.5 ± 0.500^#^

7.	Plum	20.5 ± 0.645 c^*∗*^	10.5 ± 0.288^#^	18.75 ± 0.478 d^*∗*^	10.5 ± 0.500^#^	18 ± 0.408 d^*∗*^	10.25 ± 0.478^#^	19.75 ± 0.750 c^*∗*^	11 ± 0.408^#^

8.	Pineapple	5 ± 0.408 a^*∗*^	11 ± 0.816^#^	5.75 ± 0.629 b^*∗*^	11 ± 0.816^#^	6.75 ± 0.250 ab^*∗*^	11 ± 0.707^#^	6.75 ± 0.250 a^*∗*^	12 ± 0.408^#^

9.	Guava	18.5 ± 1.32 c^*∗*^	10.25 ± 0.629^#^	19.5 ± 0.645 d^*∗*^	10.5 ± 0.288^#^	17.5 ± 0.645 d^*∗*^	9.5 ± 0.288^#^	18.75 ± 0.478 c^*∗*^	10.75 ± 0.750^#^

^*∗*^Four replicates each with *n* = 50, 25 males and 25 females (1 h @ intervals of 10 min), with total *n* = 200. Values in the table represent number of mosquito landings; ASBs with different letters (column-wise) and different symbols (row-wise) are significantly different (*p* < 0.05) computed by one-way ANOVA, followed by Tukey's all pairwise multiple comparison test.

**Table 2:** Percentage of laboratory and field strain mosquitoes attracted towards different ASBs in screening and postscreening assays.

ASBs	% mosquitoes landed on the bait			
	Laboratory strains		Field strains	
	AND*-Aedes aegypti*	AND-*Aedes aegypti-*DL10	*Aedes aegypti* (SHD-Delhi)	*Aedes aegypti* (GVD-Delhi)
Screening cage-A				
Control	4.0%	30.0%	14.0%	17.0%
Watermelon juice-ASB	10.0%	24.0%	11.0%	12.0%
Muskmelon juice-ASB	3.00%	25.0%	13.0%	13.0%
Mango juice-ASB	17.0%	34.0%	24.0%	25.0%

Screening cage-B				
Control	11.0%	24.0%	24.0%	20.0%
Papaya juice-ASB	8.0%	26.0%	14.0%	14.0%
Orange juice-ASB	15.0%	10.0%	21.0%	8.0%
Plum juice-ASB	23.0%	30.0%	39.0%	30.0%

Screening cage-C				
Control	16.0%	24.0%	17.0%	24.0%
Pineapple juice-ASB	18.0%	12.0%	10.0%	14.0%
Sweet lemon juice-ASB	14.0%	10.0%	21.0%	10.0%
Guava juice-ASB	28.0%	34.0%	31.0%	36.0%

Postscreening				
Control (10% sucrose)	16.0%	26.0%	16.0%	20.0%
Mango juice-ASB	24.0%	30.0%	17.0%	24.0%
Plum juice-ASB	31.0%	31.0%	33.0%	30.0%
Guava juice-ASB	36.0%	36.0%	37.0%	38.0%

^*∗*^Four replicates each with *n* = 50, 25 males and 25 females (1 h@ intervals of 10 min), with total *n* = 200.

**Table 3:** Relative attractant efficacy^*∗*^ of the three ASBs with respect to control against laboratory and field strains of *Aedes aegypti* in the postscreening assay.

Mosquito Strains	Fruit juice in ASB formulation			
	Control	Mango	Plum	Guava
Laboratory strains				
AND-*Aedes aegypti*	1.00	1.50	1.94	2.25
AND-*Aedes aegypti-*DL10	1.00	1.54	1.19	1.38

Field strains				
* Aedes aegypti* (SHD-Delhi)	1.00	1.06	2.06	2.31
* Aedes aegypti* (GVD-Delhi)	1.00	1.20	1.50	1.90

^*∗*^Relative attractant efficacy: mean number of mosquitoes attracted to the baits/mean number of mosquitoes attracted to the control.

## Data Availability

The data used to support the findings of this study are included within the article.
